# Transient Analysis of Fresnel Zone Plates for Ultrasound Focusing Applications

**DOI:** 10.3390/s20236824

**Published:** 2020-11-29

**Authors:** Sergio Pérez-López, Daniel Tarrazó-Serrano, Dimitry O. Dolmatov, Constanza Rubio, Pilar Candelas

**Affiliations:** 1 Centro de Tecnologías Físicas, Universitat Politècnica de València, 46022 València, Spain; serpelo1@teleco.upv.es (S.P.-L.); dtarrazo@fis.upv.es (D.T.-S.); crubiom@fis.upv.es (C.R.); 2 National Research Tomsk Polytechnic University, Tomsk 634050, Russia; dolmatovdo@tpu.ru

**Keywords:** fresnel zone plate, acoustic lens, transient analysis, temporal focusing profile

## Abstract

Fresnel Zone Plates are planar lenses that can be used to focus ultrasound beams. This kind of acoustic lenses can play a key role in the resolution of ultrasonic NDT systems. In this type of pulse-echo applications, the pulse duration is an important parameter that specifies the axial resolution, and thus, shorter ultrasound pulses provide higher resolutions. However, acoustic lenses exhibit a transient response that should be considered when setting the pulse duration, as pulses shorter than the transient state duration result in degradation in the response of acoustic lenses in terms of focal intensity, focal displacement, and lateral and axial resolutions. In this work, a thorough analysis of the transient response of Fresnel Zone Plates is discussed, demonstrating that the transient state should be considered in order to achieve optimal focusing performance. Theoretical and numerical results are presented, showing very good agreement.

## 1. Introduction

The generation of focused acoustic fields is a hot topic in different scientific and engineering fields, including pharmaceutical, sonochemistry, and nondestructive testing [1,2,3]. In recent years, different novel approaches to focus acoustic beams were proposed, including the application of metasurfaces [4], time-reversal mirrors [5], holographic lenses [6], Polyadic Cantor Fractal lenses [7], and Pinhole and Fresnel Zone Plates [8,9]. Fresnel Zone Plates (FZPs) are a very appealing option to focus acoustic waves in cases where the size, weight, and production simplicity of the acoustic lens are key constraints. FZPs are planar lenses with an easy design and manufacturing process and can be effectively employed in both air and underwater applications [10,11]. FZPs consist on a series of concentric Fresnel regions. The phase difference from the lens to the focus between two consecutive regions is π, which means that the pressure contributions between consecutive regions interfere destructively at focus. Based on this phenomenon, FZP lenses can be classified in two types, depending on how this phase change is treated: Soret FZPs and Phase-Reversal FZPs [12].

Flaw detection and sizing is one of the main applications of pulse-echo ultrasonic testing, which makes the task of focusing acoustic fields relevant. With the aim of improving the resolution of these techniques, J. Salazar et al. carried out various studies. They proposed the application of two pulses to excite the transducer (the so-called pulse cancellation technique [13]). Moreover, the same authors discussed an air-coupled ultrasonic testing system based on the pulse cancellation technique [14]. In general, acoustic focusing is obtained using transducers with concave surfaces and testing objects immersed in water. The application of FZPs has the potential to increase the versatility of ultrasonic inspections. This is related to the fact that the parameters of the focal spot (depth, length, and width) can be modified via the appropriate design of the FZP according to the objectives and conditions of the testing.

One of the basic features of pulse-echo testing is the application of pulsed ultrasonic signals. In this sense, the signal duration should be short enough to provide results with the required axial resolution. Previous studies showed that pulse compression techniques can be used to decrease the ultrasound pulse length in order to improve the axial resolution [15]. Short impulses allow for the effective solving of the aims of pulse-echo ultrasonic nondestructive testing. It is possible to reduce the size of the dead zone, increasing the resolution and precision of determining the distance to the reflector in testing objects [16]. On the other hand, using acoustic lenses requires employing excitation signals with shape and duration that result in effective focusing performance. In this regard, introducing FZPs to the pulse-echo ultrasonic testing field requires a comprehensive study on how the shape and duration of the pulsed signals affects the focusing efficiency of FZP lenses.

In this work, a thorough analysis of the transient state of FZP lenses is presented. Three waveform types have been considered, including Continuous Wave (CW), Modulated Rectangular Pulse (MRP), and Modulated Gaussian Pulse (MGP). The pulse duration influence on the main lens parameters is studied, demonstrating that if the pulse length is lower than the transient state duration of the lens, optimal resolution parameters are not achieved.

## 2. Results

### 2.1. FZP Transient Duration

FZP lenses are widely used in many application fields due to their simple design, planar fabrication, and good focusing performance. They are made of circular concentric rings with decreasing width, known as Fresnel regions. Each Fresnel region is in phase-opposition with the previous one, meaning that it generates a destructive wave interference at the focal distance of the lens. Thus, FZPs can be divided into two types depending on how they handle the phase-opposition regions. Soret FZPs block phase-opposition regions with pressure opaque rings so the resulting lens is an alternating sequence of transparent and pressure blocking rings. On the other hand, Rayleigh-Wood FZPs, also known in literature as Phase Zone Plates or Phase-Reversal FZPs, replace the blocking regions with phase-reversal rings, which introduce a π-phase shift and therefore all the Fresnel regions of the lens can contribute constructively at the focus.

The design condition of the lens is that the path difference from the radius of each contiguous region to the focus has to be λ/2, which provides the phase-opposition condition between consecutive Fresnel regions. Thus, if plane wave incidence is considered, the radius of each Fresnel region, $r_{n}$, is given by

(1) $$r_{n}=\sqrt{n\lambda F+{\left(\frac{n\lambda }{2}\right)}^{2}},$$

where λ=c/f is the wavelength, being *c* the sound speed in the medium and *f* the working frequency, *F* is the focal distance, and n=1,2,…,N, where *N* is the total number of Fresnel regions.

When a plane wavefront hits the lens, the time of flight from each region to the focus is different, as the propagation paths of outer rings are longer than that of inner rings. This means that the lens will have a transient response, starting with the arrival of the wavefront generated from the diffraction at the first Fresnel region and ending with the arrival of the wavefront diffracted from the last region of the lens. After this transient response, a steady state is achieved, as the focus includes the diffracted contributions from all Fresnel regions. Therefore, when FZPs are used in pulsed ultrasound systems, the pulse duration should be long enough to ensure that the steady state is reached or degradation on the lens resolution should be expected, as the focus will never receive simultaneously the contributions of all the rings of the lens.

In this sense, if the transient state is defined from the propagation delay point of view and the first region is a pressure blocking ring, the transient state duration, Δt, can be calculated as the difference between the time of arrival at the focus of the wavefront generated at the first radius and the wavefront generated at the last radius of the lens, that is,

(2) $$\Delta t=\frac{\sqrt{r_{N}^{2}+F^{2}}-\sqrt{r_{1}^{2}+F^{2}}}{c}=\frac{(N-1)\lambda }{2c}.$$

**Figure 1:**
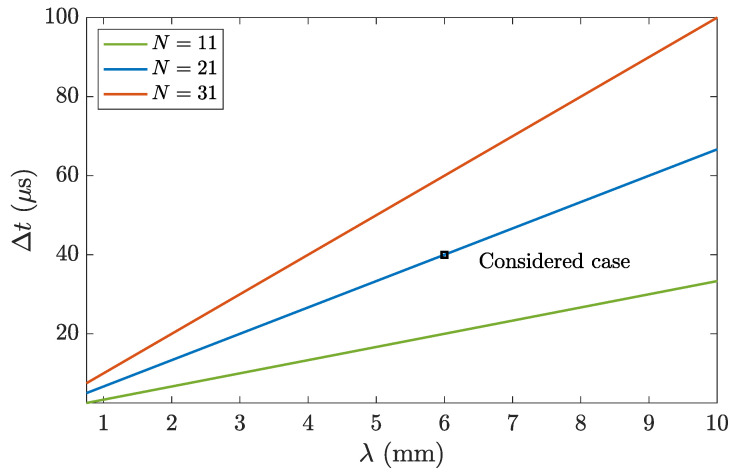
Transient state duration Δt as a function of the wavelength: N=11 (green), N=21 (blue), and N=31 (red).

Figure 1 shows the transient state duration as a function of the wavelength for three different lens sizes: N=11, N=21, and N=31. As it can be observed in the figure, the transient duration increases linearly with the wavelength, and thus higher frequency lenses will exhibit shorter transient times. Moreover, as the size of the lens increases, the transient duration increases too. It is worth noting that, as shown in Equation (2), the transient duration does not depend on the focal distance. This is a consequence of the design condition of the lens; that is, the path difference between consecutive regions is always λ/2, independently on the focal distance of the lens.

### 2.2. FZP Transient Response

In order to analyze the transient state influence on the focusing parameters of the lens, i.e., axial and lateral resolutions, focal distance and focal intensity, a Soret lens with N=21 Fresnel regions, λ=6 mm, and a focal distance of F=50 mm has been selected. A sound speed propagation of c=1500 m/s is considered in water, so λ=6 mm is achieved for a frequency of f=250 kHz. As shown in Figure 1, this lens provides a transient duration of Δt=40 μs. Three different waveform shapes have been analyzed: an ideal Continuous Wave (CW) sinusoidal signal, a Modulated Rectangular Pulse (MRP), and a Modulated Gaussian Pulse (MGP). These waveform types can be described as

(3) $$\;\mathrm{CW}:\;x\left(t\right)=x_{0}\sin\left(2\pi f_{0}t\right)$$

(4) $$\;\mathrm{MRP}:\;x\left(t\right)=x_{0}\sin\left(2\pi f_{0}t\right)\cdot \mathrm{rect}\left(\frac{t-T_{0}/2}{T_{0}}\right)$$

(5) $$\;\mathrm{MGP}:\;x\left(t\right)=x_{0}\sin\left(2\pi f_{0}t\right)\cdot e^{-\frac{{\left(t-t_{0}\right)}^{2}}{2\sigma_{t}^{2}}}$$

where $x_{0}$ is the signal amplitude, $f_{0}$ is the central frequency of the waveform, rect(·) is the rectangular function, $T_{0}$ is the duration of the rectangular pulse, $\sigma_{t}$ is the standard deviation of the Gaussian pulse, and $t_{0}$ is its offset starting time. The spectra of the three waveforms is given by the Fourier transform of the temporal signal, which results in

(6) $$\mathrm{CW}:\;X\left(f\right)=\frac{x_{0}}{2j}\left[\delta \left(f-f_{0}\right)-\delta \left(f+f_{0}\right)\right]$$

(7) $$\mathrm{MRP}:\;X\left(f\right)=\frac{x_{0}T_{0}}{2j}\left[\sin c\left(T_{0}\left(f-f_{0}\right)\right)e^{-j\pi T_{0}\left(f-f_{0}\right)}-\mathrm{sinc}\left(T_{0}\left(f+f_{0}\right)\right)e^{-j\pi T_{0}\left(f+f_{0}\right)}\right]$$

(8) $$\mathrm{MGP}:\;X\left(f\right)=\frac{x_{0}\sigma_{t}\sqrt{2\pi }}{2j}\left[e^{-2\pi^{2}\sigma_{t}^{2}{\left(f-f_{0}\right)}^{2}}e^{-j2\pi t_{0}\left(f-f_{0}\right)}-e^{-2\pi^{2}\sigma_{t}^{2}{\left(f+f_{0}\right)}^{2}}e^{-j2\pi t_{0}\left(f+f_{0}\right)}\right]$$

**Figure 2:**
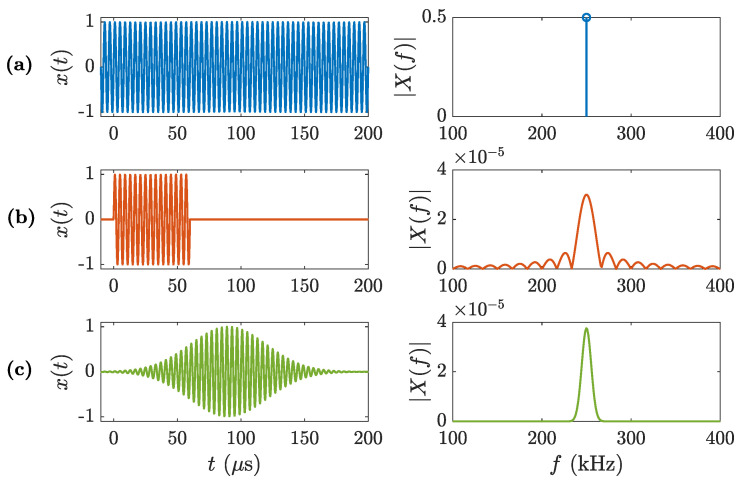
Waveforms (left) and their corresponding spectra (right): (**a**) Continuous Wave, (**b**) Modulated Rectangular Pulse of $T_{0}=60$ μs, and (**c**) Modulated Gaussian Pulse of $\sigma_{t}=30$ μs.

Figure 2 depicts the three considered waveforms in both time (left) and frequency (right) domains. The MRP duration is set to $T_{0}=60$ μs, which ensures that the steady state will be achieved as $T_{0}>\Delta t$. On the other hand, the standard deviation of the MGP is set to $\sigma_{t}=30$ μs. For this waveform case, the Gaussian pulse duration is considered to be, approximately, $T_{0}\approx 2\sigma_{t}$, which means that the MGP duration should also be enough to achieve the steady state.

The transient state of the lens is analyzed using the transversal and the longitudinal focusing profiles as a function of time. In cylindrical coordinates, the transient pressure distribution generated by the lens can be described by the pressure map |P(r,z,t)|, where *r* represents the radial axis parallel to the lens and *z* represents the longitudinal axis perpendicular to the lens. In this sense, the longitudinal focusing profile is defined as the pressure distribution along the central axis of the lens, that is, |P(z,t)|=|P(r=0,z,t)|, while the transversal focusing profile represents the pressure distribution at the focal distance, |P(r,t)|=|P(r,z=F,t)|. The transient pressure distribution, |P(r,z,t)|, has been calculated both theoretically and numerically. The theoretical approach is based on calculating the Rayleigh-Sommerfeld diffraction spectrum of the lens. In this method, the longitudinal and transversal focusing profiles are obtained in the frequency domain, |P(z,f)| and |P(r,f)|, using as input information the lens radii and the excitation waveform spectra depicted in Figure 2, and then the transient profiles |P(z,t)| and |P(r,t)| are calculated as their inverse Fourier transform. The numerical results are obtained by solving a Finite Element Method (FEM) model with a transient solver (see more details at the Methods section).

**Figure 3:**
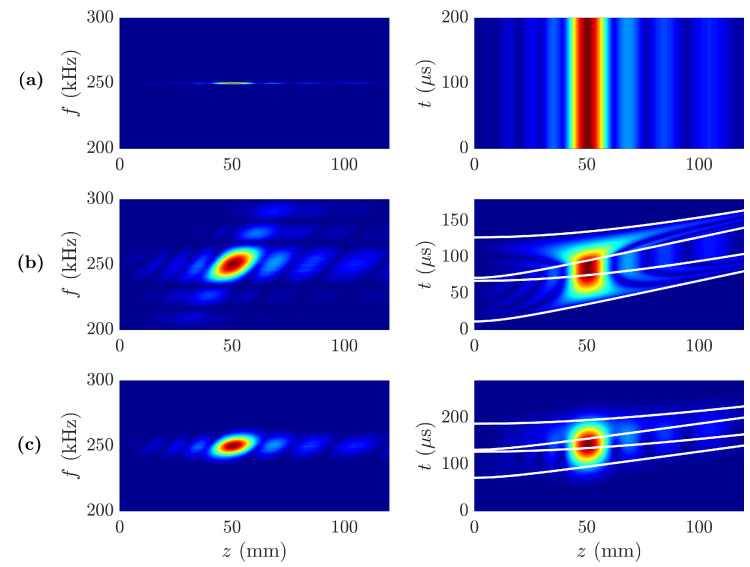
Longitudinal focusing profile spectra |P(z,f)| (left) and transient focusing profile |P(z,t)| (right): (**a**) Continuous Wave, (**b**) Modulated Rectangular Pulse, and (**c**) Modulated Gaussian Pulse.

Figure 3 depicts the longitudinal focusing profile spectra |P(z,f)| for the three different waveforms of Figure 2 and their corresponding time responses |P(z,t)|. As shown in Figure 3a, the longitudinal spectrum of the lens with CW excitation has only values for the frequency of the continuous wave $f_{0}=250$ kHz, as expected, because the frequency response of the CW is an ideal delta function centered at $f_{0}$. The time response |P(z,t)| shows no transient response, as it represents the steady state of the lens. Figure 3b depicts the longitudinal spectrum for the MRP and its temporal response. In contrast to the CW case, the longitudinal profile of the MRP exhibits a clear transient response, then reaches a steady state, and finally another transient response until the MRP propagates away from the focus. The transient states of the MRP |P(z,t)| profile show that the pressure is first focused in the focal area and then spread out over the *z*-axis. Finally, Figure 3c depicts the longitudinal spectrum of the MGP case and its time response. In this MGP case, it is worth noting that during the transient state, the pressure is more focused on the focus than in the MRP case. This means that, for the MGP case, the pressure is less dispersed along the axial distance and therefore, more spatially concentrated around the focus than for the MRP case during the transient states delimited between the first and the second, and the third and the fourth white lines of Figure 3b,c.

In Figure 3, four white lines have been superimposed over the temporal |P(z,t)| maps of the MRP and MGP cases, in order to highlight the three stage response (transient-steady-transient). From bottom to top, the first line represents the incident wavefront diffracted from the first Fresnel region as a function of time, which sets the starting point of the transient response; and the second line represents the wavefront diffracted from the last Fresnel radius of the lens, and thus it sets the ending point of the transient state and the starting point of the steady state. The position of the first line is therefore given by $t_{1}=\sqrt{r_{1}^{2}+z^{2}}/c$, while the second line is given by $t_{2}=\sqrt{r_{N}^{2}+z^{2}}/c$. Thus, for z=F the difference between the second and the first line corresponds to the transient state duration as described by Equation (2). From that moment on, the wavefronts from all the lens regions are overlapped and contribute constructively to the focus. The third line represents the last wavefront from the first Fresnel region, due to the limited duration of the pulse, and its position is calculated as the first white line plus the duration of the pulse, that is, $t_{3}=t_{1}+T_{0}$. This third line sets the ending point of the steady state and the new starting point of the transient state. Thus, the steady state duration can be calculated as the difference between the third and the second line, which for the focal distance results in $t_{3}-t_{2}=T_{0}-\Delta t$. Finally, the fourth line represents the last wavefront from the last radius of the lens, located at $t_{4}=t_{2}+T_{0}$, and sets the end of the second transient stage.

**Figure 4:**
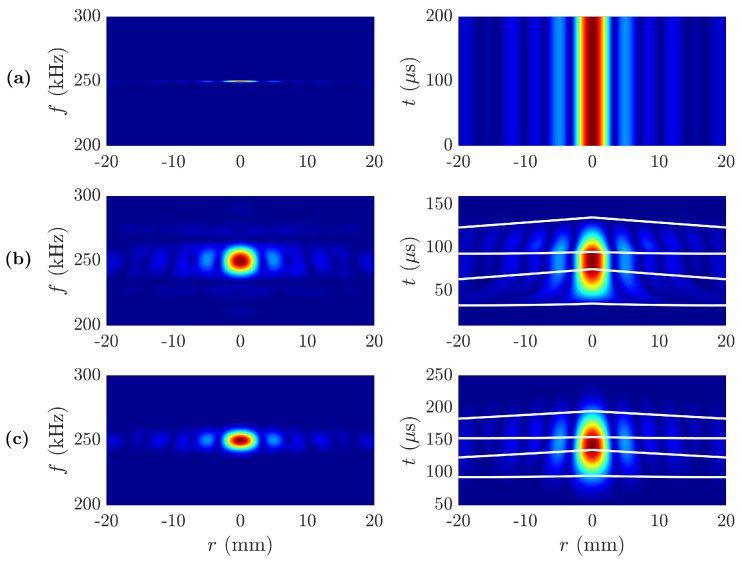
Transversal focusing profile spectra |P(r,f)| (left) and transient focusing profile |P(r,t)| (right): (**a**) Continuous Wave, (**b**) Modulated Rectangular Pulse, and (**c**) Modulated Gaussian Pulse.

Figure 4 shows the transversal focusing spectra |P(r,f)| and time responses |P(r,t)| for the same three cases of Figure 2. Similarly to Figure 3a, Figure 4a shows that for the CW case the transversal spectrum has only content at the central frequency $f_{0}=250$ kHz, and therefore the time response exhibits no transient states. On the other hand, Figure 4b,c show clear transient responses for the MRP and MGP cases, respectively. In these |P(r,t)| pressure maps, the four white lines have the same meaning as in the |P(z,t)| maps. In both Figure 4b,c, the three stage response can be observed, with an initial transient state, followed by an steady response, and then another transient state.

**Figure 5:**
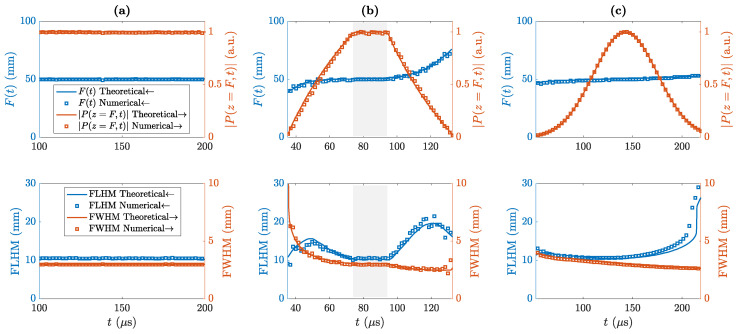
Main focusing parameters for the three different waveforms: (**a**) Continuous Wave, (**b**) Modulated Rectangular Pulse, and (**c**) Modulated Gaussian Pulse. Top plots depict the focal distance and focal intensity as a function of time, while bottom plots show FLHM and FWHM resolutions. Gray areas in (**b**) represent the transient state.

Finally, the main focusing parameters have been analyzed. These parameters include: the pressure at the focal distance, the focal distance, the Full Length at Half Maximum (FLHM), and the Full Width at Half Maximum (FWHM). The FLHM specifies the lens resolution along the *z*-axis, while the FWHM specifies the resolution along the *r*-axis. Thus, Figure 5 depicts these four parameters as a function of time for the three considered waveforms. Solid lines represent theoretical results, whereas squares represent numerical results obtained using the FEM model. As can be observed in the figure, numerical and theoretical results are in very good agreement. The steady state parameters, obtained using the CW results depicted in Figure 5a, result in a focal distance of F=50 mm, an axial resolution of FLHM =10.3 mm, and a lateral resolution of FWHM =3.0 mm. In the MRP case, depicted in Figure 5b, the focal distance increases with the focal pressure, then reaches the steady state at its theoretical focal distance F=50 mm with a sustained focal pressure, and finally increases again in the second transient state as the focal pressure decreases. The resolutions also show a first transient stage, then reach their steady resolutions (same as in the CW case), and finally another transient state. It is worth noting that, in the case of the lateral resolution (FWHM), the resolution increases at the first transient state (lower FWHM values) and also increases at the second transient state beyond its steady value of FWHM =3 mm. This happens because the first transient is caused by the wavefronts generated at the inner Fresnel regions, which contain the lowest spatial frequencies (widest regions) and therefore, low lateral resolution information, while the second transient is generated by the outer Fresnel regions, which carry the highest spatial frequencies (narrowest regions) and therefore, high lateral resolution information. The steady state duration, which can be directly calculated either from the FLHM plot of the focal pressure plot in the MRP case, is approximately 20 μs, which agrees with the theoretical calculation of the pulse duration minus the transient state duration, $T_{0}-\Delta t=60-40=20$ μs. Finally, the MGP waveform FZP response is shown at Figure 5c. In this case, the transient state is not clearly visible. This phenomenon is a consequence of the Gaussian shape of the waveform, which means that the pulses diffracted at the different Fresnel regions will not overlap exactly with the same amplitude, as each pulse has a different time of arrival to the focus, and therefore no steady focal pressure level can be achieved. This fact is also the reason why the steady FLHM is never achieved (CW case), and the MGP provides a maximum axial resolution of FLHM =10.5 mm. However, as the MGP duration is long enough to cover the transient response state ($2\sigma_{t}=60$ μs >Δt), the axial resolution is only reduced by 0.2 mm (≈0.03λ), which is an acceptable degradation. As can be observed from the figure, the focal pressure has a Gaussian shape and the focal distance increases linearly as a function of time, reaching the theoretical focal distance of F=50 mm around the maximum focal pressure levels. The steady lateral resolution FWHM =3 mm is achieved around the maximum pressure value too and, as in the MRP case, the lateral resolution increases with time due to outer Fresnel regions carrying higher spatial frequencies than inner regions. Therefore, in both MRP and MGP waveform cases, if the duration of the pulse had been shorter than the transient duration Δt, the main focusing parameters of the lens would have been distorted: shifted focal distance, reduced focal pressure, and degraded axial and lateral resolutions.

## 3. Methods

### 3.1. Rayleigh-Sommerfeld Spectrum

The theoretical analysis of the FZP transient response is based on a two step process. First, the FZP response is calculated in the frequency domain using the Rayleigh-Sommerfeld integral, and then the transient response is calculated as an inverse Fourier transform. The Rayleigh-Sommerfeld diffraction integral is given by

(9) $$P\left(r,z,f\right)=\frac{X(f)}{j\lambda }\int_{0}^{2\pi }\int_{0}^{r_{N}}t\left(\rho \right)\frac{e^{-jkR}}{R}\rho \cos\theta d\rho d\varphi ,$$

where X(f) is the waveform spectrum, k=2πf/c is the wavenumber, (ρ,φ) are the radial and angular coordinates over the lens surface, $R=\sqrt{z^{2}+r^{2}+\rho^{2}-2r\rho \cos\varphi }$, and cosθ=z/R. The function t(ρ) represents the transmission function of the lens, which for a Soret FZP type is 1 at the transparent Fresnel regions and 0 at the pressure blocking regions. Once the focusing spectrum is calculated, the transient response can be finally calculated as an inverse Fourier transform:

(10) $$P\left(r,z,t\right)=\int_{-\infty }^{+\infty }P\left(r,z,f\right)e^{j2\pi ft}df.$$

Equations (9) and (10) are both computed in MATLAB.

### 3.2. Numerical Model

The numerical model is based on a Finite Element Method (FEM) simulation using COMSOL Multiphysics 5.5. The model is solved in the time domain using a transient solver. The FZP lens is modeled as an axis-symmetric model, with pressure conditions in the transparent Fresnel regions and sound hard boundary conditions at the pressure blocking regions. The pressure condition is set to the corresponding time-dependent waveform function under study. The water domain, modeled with density $\rho_{0}=1000$ kg/m${}^{3}$ and sound speed $c_{0}=1500$ m/s, is surrounded by Perfectly Matched Layers (PMLs) in order to avoid reflections. The time-step of the solver is set to $1/(60f_{max})$, being $f_{max}=f_{0}=250$ kHz the maximum frequency present in the model, to ensure convergence of the solution. The maximum mesh size is fixed to $\lambda_{0}/6=1$ mm. After the solution is computed, the results are exported as a text file and then processed in MATLAB to obtain the main focusing parameters variation as a function of time.

## 4. Conclusions

Pulsed ultrasonic systems are widely used in NDT applications. In this kind of scenario, the pulse duration is an important parameter that is used to control the axial resolution. This resolution can be further increased with passive ultrasonic lenses, such as FZPs. In this work, a transient analysis of FZP lenses is presented using both theoretical and numerical results, demonstrating that if the pulse duration is shorter than the transient state duration, degradation in both lateral and axial resolutions is observed. It is worth noting that, although this work is focused on FZPs, the same study could be carried out for other kind of ultrasound lenses, and a similar three stage transient-steady-transient response should be expected. Particularly, when planar lenses or 3D printed lenses attached to flat transducers are used, the propagation delay from the lens to the focal distance depends on the position along the lens surface, which will produce a transient response.
